# Mr. Charles Bell's Treatise on the Urethra, Vesica Urinaria, Prostate, and Rectum, with Notes by Mr. John Shaw, Demonstrator of Anatomy, &c. &c.

**Published:** 1820-09-01

**Authors:** 


### VIII.

*A Treatise on the Urethra, Vesica Urinaria, Prostate, and Rectum.* By CHARLES BELL, Surgeon to the Middlesex Hospital, Lecturer on Anatomy in the School of Great Windmill Street. A new Edition, with Notes, containing the Criticisms of the Editors of the foreign Editions, and the Opinions of foreign Authors on these Diseases, by JOHN SHAW, Surgeon; Demonstrator of Anatomy in the School of Great Windmill Street. One Vol. Octavo, pp. 416, with five Plates. London, 1820.

THIS new Edition may be considered almost a new work; and when we reflect on the talents of the author, the industry of his Annotator, and the opportunities which both have had, of prosecuting a pathological investigation of the diseases above mentioned, we are led to expect a work of great practical merit from their hands; nor in that expectation will the public be disappointed. The author wisely throws himself completely upon his profession.

"There is here no new method of curing stricture proposed; no multiplied cases of success, until the reader must suppose him infallible; no cases to suit the feelings of alarmed patients. On the contrary, he has dwelt only on the difficulties of practice, and has exhibited, with all the force he could command, the dangers we run by rashness." xx.

This is as it should be. Talent and application have no occasion to descend to those paltry manœuvres, in which such swarms of needy and greedy adventurers are exercising themselves by day and by night, to gain a temporary PHOSPHORESCENCE which, like that surrounding a decomposing carcase in the dark, is dispelled by the first beams of the rising sun, and exposes to view the disgusting source of the mephitic radiance.

Mr. Shaw opens the work with some accurate and ingenious observations on the anatomy of certain parts of the urethra, and about the neck of the bladder, to which we cannot do justice in an analysis. He maintains, with Mr. Bell, the non-muscularity of the mucous membrane of the urethra; and affirms, that the appearance of circular muscular fibres, which anatomists have described as existing in the membrane of the urethra of the larger animals, is caused by the small vessels running on the outside of the membrane. It is not to be expected that we can decide on these nice points of anatomy; but, as Mr. Shaw admits that "the mucous membrane, though very thin, is elastic," and has the power of *contractility*, we do not see how it materially affects our practice, whether this contraction result from the action of muscular or of any other kind of fibres. But more of this hereafter. It ought to be practically borne in mind, that the mucous membrane of the urethra may be traced, not only along the internal surface of the bladder, but along the ureters to the pelvis of the kidneys; and, in another direction, from the urethra along the seminal ducts, through the convoluted tubes of the testicle, and into the vesiculæ seminales.

"Thus we can trace an inflammation of the urethra by continuous sympathy into the bladder, producing irritable bladder, into the kidneys, causing nephritis, and along the vas deferens, producing swelled testicle; each of these consequences being as likely to happen from irritation of the urethra, as the inflammation of the mucous membrane of the nose is, in common catarrh, to proceed along the membrane of the trachea into the lungs." 5.

There are some local peculiarities of sympathy here also, which are worthy of being kept in mind: thus, when a bougie enters the neck of an inflamed bladder, it often happens that the patient does not complain of pain at *that part*, but refers it to the glans, and thinks the surgeon holds the glans too tightly. This sympathy is well exemplified in calculus of the bladder. Here Mr. Shaw introduces many excellent anatomical remarks, and two figures, illustrative of the inequalities of diameter and dilatability of different parts of the urethra, which the surgeon should carefully study. Speaking of the resistance which the circular ligament, posterior to the bulb of the urethra, often gives to the introduction of the catheter or bougie, Mr. Shaw makes the following melancholy reflection, which we fear is too well founded!

"Dissection leads us to suspect that strictures are more frequently made at this part by the surgeons instruments, than by the effect of inflammation from common irritation."

It has long been a rule to examine the urethra with a large in preference to a small bougie; but it must be recollected, that the sinus pocularis, a lacuna in the posterior part of the canal, at the beginning of the caput Gallinaginis, will admit the point of a bougie of the size of No. 5; and in cases of stricture it is larger than in a healthy urethra.

"When a bougie enters it, the pain is so exceedingly acute, that the patient springs back, even though the rest of the canal may not be at all irritable."

Indeed, dissection shows it as a matter of constancy, that whenever there has been irritation of any kind in the urethra or bladder, the ducts of the prostate will be found enlarged, "some of them to such a degree as to admit the point of the largest bougie."

The first chapter of the work contains many interesting observations on "the sensibility of the bladder, and some of its morbid affections." The most irritable point on the internal surface of this receptacle, is directly over the junction of the muscles of the ureters with the internal sphincters of the bladder; and here the membrane will be found more vascular than at any other part. "Here is the seat of that sensibility which governs the action of the muscles." When the stone rests here, the patient suffers great pain, with an unceasing *nisus* to make water.

"It will sometimes occur, that the inflammation originally seated in the urethra, spreads backward to this spot, and then the call to make water is incessant; and by the incessant call to action, a general inflammation of the bladder is at length produced." 13.

We cannot follow Mr. Bell and Mr. Shaw in their important remarks on the action of the muscles surrounding the neck of the bladder and posterior part of the urethra; but pass on to a subject by no means beneath the dignity of the practical surgeon—*incontinence of urine.* Every one knows how unpleasant it is for children, and still more so for grown people, to make water in bed.

"This occurrence never takes place, but when the boy is asleep upon his back; and the cure is a simple one: he is to accustom himself to sleep upon his face or side; the urine is not passed, nor is he excited to dream of making urine while he keeps this position.

"The circumstance is unaccountable, until we reflect on the position of this master spring of the muscles of the bladder; the sensible spot a little behind and below the orifice of the bladder. When a person lies upon his belly, the urine gravitates towards the fundus; but when he lies on his back, it presses upon this sensible spot, and distends that part of the bladder which is towards the rectum." 18.

When a child wets the bed, Mr. Bell thinks it is in consequence of a dream, excited by the irritation of this sensible spot of the bladder, the urine resting there and stretching the bladder. "If the child be made to lie on the belly, or inclining to that position, with the cheek upon the pillow, the habit will be broken." A more serious incontinence, however, in boys, is sometimes the result of inflammation in the bladder, accompanied by pain and all the symptoms of stone. Stillicidium urinæ, from want of action in the sphincter muscles, is generally owing to an increased sensibility in the part of the bladder above mentioned.

As inflammation of the mucous membrane of the fauces will cause suppuration in the arches of the throat, and a long-continued inflammation in the membrane of the larynx, very dangerous suppurations about the cartilages of the larynx; so protracted irritation about the anus will produce abscess and fistula there. The natural susceptibility of the spot behind the orifice of the bladder is very great; and, in its morbid derangement, exquisite. The consequences of abscesses around the prostate gland and vesiculæ seminales are always exceedingly distressing.

"This disease is marked by frequent and painful calls to make urine; by a burning sensation and violent spasms after the urine has been discharged. There is, also, pain in the extremity of the penis as in stone. It is attended with a purulent discharge from the urethra, not continually and in small quantities, but at irregular periods and copiously. The patient is subject to cold shivering and fever; and he is pale, harassed, and wasted. On introducing a bougie, there is violent pain as it enters the neck of the bladder, and it comes out smeared with matter, and, perhaps, bloody matter. On examining per anum, a thickening is felt around the prostate gland or vesiculæ; and the patient experiences pain when you press against the part.

"The causes of this complaint are violent inflammations in the urethra, aggravated by free living and debauchery, and by irritating injections, the unskilful use of bougies, caustic, and cantharides: but most of all, I fear it, in a scrofulous constitution." 21.

*Treatment.* We must lessen the irritability and diminish the vascular action of the part—dilute and change the quality of the urine. The means are, laxatives; the application of leeches to the anus; emollient and anodyne glysters; mucilaginous drinks, as infusions of linseed, with alkalies and opiates; but all these are inferior in efficacy to *injection of the bladder*.

This operation had not been favourably received by British surgeons; yet Mr. Bell and others,* have found it very useful in relieving irritation at the neck of the bladder. When

---

* See our Review of Mr. Wadd's Works, No. 8, Quarterly Series.

this state accompanies stricture of the urethra, the bougie is a dangerous instrument, especially in the hands of the patient. " But it is sufficient on these occasions to throw up a little tepid water into the urethra. The presence of the injection brings on the consent of parts, and is followed by discharge of water, with relief." In fits of the stone the injection of tepid water distends the bladder, and removes the calculus from the morbidly sensible spot near its neck. " If two or three ounces of fluid be very slowly injected into the bladder, the excess of pain will be immediately mitigated."

But it is in the case of inflammation and irritation within the neck of the bladder that this injection is of the most essential service. By gradually increasing the quantum of injected fluid, we often relieve the pain, and detect calculi that could not otherwise be felt by the sound. During the past year Mr. Bell employed the remedy in five cases.

" The first of these was a dwarfish boy, who was brought into the hospital with the suspicion of stone: he was sounded twice, and no stone discovered. Some months after, he returned with the same complaint; a painful and frequent call to make water, with pain in the extremity of the penis. He was put under the charge of a dresser, to have the bladder injected. He expressed himself relieved from the first; gradually more and more water was admitted into the bladder; every day the bladder could contain an increased quantity of water; and, after some weeks, he was dismissed well." 24.

Above all, Mr. Bell recommends the injection of the bladder in the commencement of the disease called " uvula vesicæ." When there is an inordinate irritation of the sensible spot at the neck of the bladder, nothing is so likely to allay the irritation. The rationale is obvious enough. We soothe the irritability of the part by substituting the tepid water for the acrid urine, and by diluting the latter, as it secretes from the kidneys. " Besides, by this injection of the bladder, the ropy mucus and the purulent secretion, when there is any, are washed away, and that sort of tenesmus vesicæ, caused by their presence, is relieved."

The second chapter is very valuable, and greatly enriched by the judicious notes collected by Mr. Shaw, from foreign authorities. It is on the cases requiring the aid of instruments to draw off the urine. These are, paralysis of the bladder; spasm and irregular action of the muscles at the neck of the bladder; injuries committed on the perineum by falls, kicks, &c.; diseases of the rectum; the plugging up of the urethra by small calculi, &c.; extravasation of blood compressing the urethra; disease of the prostate gland; stricture of the urethra.

Under the first head, our author introduces many important observations and reflections relative to the loss of tone in the bladder, from too long retention of the urine; and also on the necessity of attending to a case of this kind for some time after you draw off the water by the catheter, as the retention is apt to recur again and again.

Speaking of suppression of urine from spasm and irregular action of the muscles at the neck of the bladder, Mr. Bell observes:

"It is said, that in such a case we are not immediately to introduce the catheter; but that we ought to bleed the patient, and apply leeches to the perineum, and fomentations, or put him into the warm bath. But I must confess, when I have seen a man in indescribable agony, moving about my room, with his body bent to an angle, and when I have understood that before this attack his urethra was free, and that now the bladder can be distinguished above the pubis, I have followed the dictates of common sense and common charity, I have done that, which I knew would immediately relieve him, and with perfect safety: I have introduced the catheter, and have drawn off the urine." 38.

When, indeed, the retention is owing to a stricture in the urethra, then we must be cautious in the use of instruments; for if the attempt does not succeed, the symptoms are certainly aggravated. We are, in such cases, to use all the palliative means to remove the spasm, and restore the stream of urine. These means are, local and general bleeding, especially leeches to the anus and cupping the loins. When the pulse is reduced by bleeding, the warm bath, particularly the hip-bath, is useful, or even fomentations, with large glysters of tepid water and oil. After procuring stools, the opiate enema, made with starch or barley decoction, and fifty drops of laudanum, will relieve the grinding pain and irritation. These means may be followed with Dover's powder and the tepid bath, tepid diluent drinks, and a mixture with laudanum, æther, and liq. potassæ. If these means fail, what are we to do? If the state of the urethra be such as to make the introduction of the catheter improper, the simple bougie may be used; not to draw off the urine, but to bring back the muscles to their natural action.

"The patient should be standing, or resting on his knees, if he is in bed. Take a wax bougie, oil it, soften it, give it the proper curve to pass the turn of the urethra, introduce it into the bladder; now make gentle pressure above the bubes; make the patient exert himself to discharge the urine; sprinkle cold water on his thighs; withdraw the bougie while he continues the effort; and when he has the sensation as if he could pass the urine, withdraw the bougie altogether, and the urine will probably flow." 43.

Even if the stricture be such as to prevent the bougie or catheter from reaching the bladder, the patient may often be relieved by pressing gently forward with a small bougie, till the point of it has moulded or wedged itself into the stricture, when the belly is to be gently pressed, and the patient directed to exert himself to pass the urine. To further this object, his hands are to be put into cold water, or the same sprinkled on his thighs, slowly withdrawing the bougie, when the urine will generally follow. If this fails, the small catheter must be tried; and if we are foiled in this also, it is evident that we must puncture the bladder, an operation hereafter to be described.

On the introduction of the catheter Mr. Bell gives most excellent directions, which the young student should have engraven on his memory. The following *caustic* observation on the caustic bougie of this country, and *Sonde conique D'Argent* of our French brethren, is in Mr. Bell's peculiar style.

"It will be admitted that the English surgeons are very bold; and if there be a method which promises to be a shorter road to success, that they will take at all hazards. There is not a young man who does not prefer the caustic to all other means of relieving the bladder; and if it shall be proposed to destroy the stricture at once, by breaking it down, with what avidity does he seize the occasion! hence those iron pokers imported, they say, from France, which ornament the window of my neighbour, Mr. Thomson, and which they call bougies! Advantage was never gained by violence in this class of complaints." 50.

Here Mr. Bell makes many good remarks on affections of the prostate gland, which he divides into two classes:—1st. Those inflammatory affections resulting from irritation of the neighbouring parts, common to all periods of life; and, 2dly, those formidable diseases of the gland, to which the latter stages of our existence render us peculiarly liable.

"The disorders under the *first* head are very frequent, and produce great distress. We frequently follow the progress of a case in this manner: a patient, after a tedious and imperfectly cured attack of gonorrhœa, will occasionally have a copious discharge of purulent matter—occasionally, because the discharge is not as from gonorrhœa; it cannot be squeezed from the urethra: it does not flow regularly: it is not every morning that it is found to cover the linen. There is great irritation about the neck of the bladder, frequent call to make water, sometimes pain in the glans penis. If the bougie should be introduced, it is attended with a burning sensation when passing the neck of the bladder; and when it is withdrawn, much bloody mucus and pus follows. If the prostate gland be examined per anum, it is full, one lobe feels much larger than the other, and there is an induration and irregular hardness perceptible in the seat of the prostate gland and vesiculæ seminales." 51.

These effects, Mr. Bell thinks, spring from a gonorrhœa of the ducts of the prostate; the inflammation, originally seated in the membrane of the urethra, having spread back to them, the gland itself becoming inflamed, while suppurations are established in the surrounding cellular substance. "It is by thrusting the point of the instrument into the prostate, and opening these bags of matter, that the sudden and copious discharge of pus and bloody mucus follows." This, Mr. B. believes to be one of the evils following gonorrhœa in a scrofulous constitution.

"The cure of this distressing disease is to be accomplished by persevering in all the means of allaying irritation. The bowels must be kept easy and natural. The occasional attack of irritation must be soothed by opiate clysters and suppositories, the warm bath, and by mucilaginous drinks, and by leeches applied occasionally to the anus. Here I have found the injection of the bladder to be of the greatest advantage, and also friction on the part, with ointment introduced through the anus upon the point of the patient's finger." P. 52.

As this state of the neck of the bladder is attended with burning and irritation, and, occasionally, with spasm and difficulty of making water, so we must be exceedingly cautious in introducing the catheter, since the ducts of the prostate are enlarged, and the point of the instrument is apt to fall into them, or into the abscesses around.

The scirrhous and enlarged state of the prostate gland, in advanced life, is preceded by much uncomfortable feeling in the pelvis, rectum, and bladder, accompanied by sympathetic pains in the hips, perineum, and pubes. The fæces are passed with some difficulty, and the figured stool is flattened. The patient thinks he expels the last drop of urine when there is a pint or more remaining in the bladder, as ascertained by the introduction of the catheter. The gland may be equally or partially enlarged, and some idea of its condition may be formed by an examination per anum. In true disease of the prostate, the patient does not complain so much of pain at the moment we press the gland, as some time afterwards.

It ought to be borne in mind, that any thing lodging in the rectum will closely imitate disorder of the bladder; therefore, the bowels should be moved, and the fæces softened by a mild laxative, and a large glyster of tepid water every morning. A pill composed of pil. hyd. ex. conii, and ex. col. comp. will be found useful. The Dover's powder also conduces to allay the inflammatory action. The patient should be cupped upon the loins, or have leeches to the anus, with the tepid hip-bath, and afterwards an opiate glyster or suppository. The catheter must be carefully used to empty the bladder, and tepid water should be injected into this organ to soothe its irritation. The large gum catheter is the best.

The third chapter is equally interesting to the physician and surgeon. It is on pain and irritation of the urinary organs from disorder of the bowels.

"Those sympathetic pains which seem to effect the urinary organs, and which have their real seat in irritation of the rectum, or the other intestines, bring full one half of those patients to the surgeon, who are considered as labouring under stricture of the urethra. How this subject should make no part of those numerous Treatises on Stricture we possess, is very remarkable, unless we are to suppose that experience follows, and does not precede, those publications. It is a fact that ought never to be absent from the surgeon's consideration, when a patient presents himself, complaining of frequent micturition, pain in the bladder, and pain in the perineum, that these symptoms very frequently depend, neither on stone, nor stricture, nor inflammation, nor any mischief in these parts, but on remote irritation." 60.

Mr. Bell justly observes, that there is a class of patients, whose complaints are considered imaginary by those medical gentlemen, who are not inaptly said to have "a run of business." A patient came to Mr. Bell, after having been under four surgeons successively, for the cure of stricture in the urethra. Mr. B. found his chief complaint to be an excessive tenderness in the perineum, embarrassing him while he walked. He had been a fox-hunter, but for a long time past had been unable to mount a horse. The introduction of a bougie shewed no obstruction there, nor was there any unusual tenderness in the passage. He was also examined per anum; and it was remarkable, that in putting him into the posture of lithotomy, and while fingering, kneading, and pressing the perineum, he was not sensible of any pain, although, when he rose to walk, his progression was interrupted. Mr. B. was convinced that it was a pain referable to the perineum, but not actually seated there. By attention to the bowels he was relieved, so as to resume his horse exercise. Another patient, whenever by confinement to the house his bowels became torpid, experienced a sensation of pain within the anus, and at the neck of the bladder, immediately after passing fæces. This was quickly followed by a pain, as of a sharp instrument driven from behind along the urethra, and giving the gland repeated darts. To this succeeds an intolerable spasm, with an attempt to pass more water. These symptoms are not relieved till all the parts are bathed with warm water. By accident this patient discovered that wine, instead of aggravating, mitigated these symptoms. Purging increased the irritation; but balsam copaiba, which acted as a gentle laxative, soon removed the complaint. Many cases are related of anomalous symptoms and irritations of the urinary organs being relieved and dispersed by acting on the intestinal canal and biliary secretion; for which we must refer to the work itself.

*Treatment.* "The violent operation of purgatives is to be avoided. The combination of laxatives is better: thus, after emptying the canal, with the oleum ricini and tinctura sennæ, preserve the intestinal surface in activity by combinations of ipecacuanha, pulvis rhei, and pulvis cretæ cum opio; or a combination of the pulvis antimonialis with the pulvis rhei, and the extractum papaveris albi; or, it may be, that it will suit better to give the electuarium sennæ with sulphur, or sharpened with the addition of jalap and oleum ricini. It may be necessary to combine opium with the oleum ricini, when there is much pain and spasm; or to add hyoscyamus to a pill of soap and extract of colocynth. Superior to all, in some constitutions, is a tea-spoonful or two tea-spoonfuls of the balsam of copaiba taken at night. When, by such means, the canal is disposed to a gentle action, let the morning evacuation be assisted by a large clyster of warm water. Very often, in these conditions of the viscera, there is only something wrong in the diet, and the symptoms will vanish, by avoiding what harbours, and is offensive. We shall find it often impossible to restore to the bowels their permanent healthy action, without stirring up the liver to its office. What I have found of most advantage, is a pill at night of three grains of the pil. hydrargyri, and two grains of the compound extract of colocynth; and in the morning, the patient may take a very small portion of neutral salts in solution, so as still to avoid purging, but only gently and regularly to move the intestines, or the carbonate of soda and tartrite of soda in a state of effervescence, with the citric acid." 76.

In the above cases, glysters of warm water, during the paroxysm, are very soothing, and go directly to the seat of irritation. "The glyster of cold water is also advantageous." Anodyne enemata or opiate suppositories will naturally be suggested in the violence of the paroxysm, with regulated diet, air, and exercise.

From the beginning of chap. iv. page 77 to page 294, the subject of stricture, with all its various consequences on the bladder, urethra, &c. is most ably discussed and illustrated by Mr. Bell and Mr. Shaw. This great division of the work embraces such a mass, and such a multiplicity of important subjects and excellent observations, that we found ourselves totally incapable of giving any thing like even a faint idea of it. It ought to be constantly at hand for reference, in all cases where the urinary organs are engaged. We shall therefore pass it over in toto, and endeavour to give some account of that portion of our author's work which treats "of diseases of the rectum," especially as this subject forms a considerable item of investigation in the present Number of the Journal.*

In this division of the work, as in the former, our author has drawn his principles from the natural and morbid structure of parts, concealing nothing, but disclosing all that he thinks important.

"Nothing tends so effectually to disorder the connexion of the sphincter and levator muscles, in the act of expelling the fæces, as the absorption of the fluids of the rectum, and the lodgment of the hard fæces in the lower part of the intestine. Then it is that the patient may strain ineffectually, for the rectum does not contract, the sphincter does not relax, the levator resists, and the only effect produced is the distention of the veins of the verge of the anus, and the forcing of the upper part of the rectum to descend into the lower and more capacious portion." 299.

The levator and sphincter ani muscles are not antagonists, but coadjutors. Their conjoint operation prevents prolapsus of the gut in the act of discharging fæces; one contracting around the extremity of the gut, the other drawing it up and sustaining it.

"But if any peculiar irritation takes place within the rectum, by which the relaxation of these muscles is excited, they lose their guardian office, and let the inner membrane of the rectum descend, and permit the veins of the anus to be distended." 300.

1. *Warts within the verge of the Anus.* This disease is sometimes mistaken for cancer, and leads to negligent or too severe practice. The patient complains of continual irritation and discharge from the anus, with difficult evacuation of the fæces, which have a ragged appearance from passing through the irregular orifice. The introduction of the finger into the anus is difficult, and draws blood; there is a certain degree of stricture at the orifice; the surface is felt hard and rough, owing to the existence of warty excrescences. These should be snipped off with probe-pointed scissars, the remaining lesser ones being touched with caustic,, or washed by means of a small sponge attached to a probe, with the diluted acetic acid. The rectum bougie is to be used. This

---

* See our Review of Mr. White's work in this Number.

disease, like most others of the rectum, will be found to have a connexion with deranged action of the bowels.

2. *Stricture of the Orifice of the Rectum.* This is by no means an uncommon complaint. It is known by the distress and pain on going to stool, by the occasional retention of the fæces, and by the form of them.

" On inspection, it is ascertained by a distinct ring prominent around the orifice, and to be felt by the finger pressed against the perineum; by the difficulty of introducing the finger, and by the unusual pain which the attempt creates.

" This stricture is occasioned by inflammation, repeatedly produced, though in a slight degree. Its cause, for the most part, is costiveness and straining, by which the fibres are strained and burst. Sometimes, I believe, it may come from tenesmus, and frequent excitement of the orifice by painful and ineffectual calls to evacuations." 303.

Stricture of the orifice, with piles, is a still more common occurrence. Thus, if a person is irregular in the time of his evacuations, and the propulsion of the fæces be attended with straining and pain; and this state continue long, a stricture of the anus is a pretty sure consequence. Mr. Bell justly remarks, however, " that through the whole length of the intestinal canal, there is a connexion betwixt the state of secretion from the inner surface, and the state of contraction of the muscular coat of the intestine."

" The cure of the stricture of the anus is to be accomplished, 1. By leeching and fomentation, to subdue the existing inflammation; 2. by correcting that state of irregularity of the bowels, which has been the original cause; 3. by the use of clysters; 4. by the introduction of the rectum bougie.

" Patients in this country, will object to the use of the *lavement.* Then let them attend to their diet, and study regularity in their evacuations; but if, by neglect of these matters, a disease of the rectum or anus comes on, there is nothing more conducive to ease and rapid recovery, than the proper use of clysters." 305.

Mr. Bell thinks there are few diseases of the anus and rectum that will not yield to the proper use of the rectum bougie. For common cases Mr. Bell prefers the metallic bougies. For a stricture of the rectum, some way within the orifice, and attended with spasm and pain, the common bougie produces distressing symptoms. A simple tent of rolled linen is more beneficial.

" Take a piece of linen of square form, roll it up in the form of a bougie, then tie a cord or strong thread very firmly around one end of it. A probe is now to be passed up in the inside of the roll of linen, until its point is stopped by the tying. Where the cloth projects beyond the ligature, it is to be cut and rounded, so as to offer no obstruction when introduced into the rectum. The tent thus formed, is to be dipped in liniment or oil; and is ready for use, The probe gives it stiffness, so as to enable us to pass it through the stricture; and the probe being withdrawn, the tent lies soft and pliant in the rectum." 307.

Here Mr. Bell recommends a dilatation of the parts by the introduction of a gut, six or eight inches long, into the rectum, and afterwards slowly distended with fluid by means of a syringe. This is similar in principle to Mr. Arnott's dilator, which we have lately found very useful in a stricture of the rectum. The bougie or dilator should be used in the morning, while the patient is still in bed; and the size, at first, should be only such as may put the parts gently on the stretch; but no violence should ever be used. If the patient can bear the instrument, it may be allowed to remain twenty minutes. Sometimes, without the patient's feeling pain, the bougie may be distending the stricture too rapidly, and doing much mischief. In these cases, a shivering and sickness follow, in a few hours. "It is possible to bring on inflammation of the peritoneum and death by this kind of violence."

"Let the patient be placed on his knees in bed; the bougie is warmed and oiled, though a mild liniment or ointment is better. It is at first introduced perpendicular to the orifice; but when about an inch within the anus, it is to be directed upward, that is, towards the sacrum: if it be far introduced, the direction must be again varied, otherwise the point will hit the promontory of the sacrum; and often this resistance of the bone has been mistaken for stricture." 309.

Mr. Bell introduces here some important observations on sounding the rectum. Obstructions may take place in this gut from various causes; from simple stricture, hardened fæces, the falling down of a turn of the colon, a tumour, as of the ovarium, or a retroversion of the womb. The history of the case should always be minutely inquired into, before a bougie be used. Mr. Bell was called to a lady who had been three years under the use of the bougie. Mr. B. "found that the obstruction to the rectum arose from the fundus of the uterus having fallen into the hollow of the sacrum." Against this had the bougie been pushed for years, fortunately without any other bad consequence than the expensive attendance of a surgeon. In the introduction of instruments into the rectum, beyond the reach of the finger, attention to the curve and form of the canal is particularly necessary; otherwise, the extremity of the instrument will be driven against the bladder, or against the sacrum.

3. *Stricture of the Rectum.* This our author considers to be owing to a morbid change in the inner membrane of the intestine. "Not unfrequently the inner edge of the deeper sphincter ani is the seat of this stricture; and then the finger enters only to the depth of the second joint, when it is obstructed by a sort of membrane standing across the passage." The symptoms of stricture are sometimes very obscure, and arrive at a great height before they are noticed by either patient or practitioner.

"Without any definite notion of the nature of the disease, the patient is, nevertheless, sensible that there is something wrong. He has spasms of the gut; he has pain of the loins, sometimes of the hips and thighs; he is distressed with flatulence and eructation; he is sensible of something obstructing the freedom of evacuation; he says his fæces are like those of a child, small and flattened, or irregularly figured. He strains much, and hence piles are often combined with stricture of the rectum, as with stricture of the urethra. The state of the bowels is altogether irregular; and not unfrequently, there is purging alternating with constipation." 312.

But there is more than this, in the condition of a patient with a narrow stricture. The obstruction to the regular discharge of fæces produces a gradual accumulation in the bowels, with prominence of the abdomen, borborigmi, and great distress. The patient now takes opening medicines, the fæces are softened, large quantities are discharged, and the patient, after extreme debility, feels himself relieved for a time, till a gradual, and at length enormous accumulation takes place in the intestines. The mind and body are again oppressed; there is faintness and lowness; the breathing is laborious; the flatus distressing; the face is pale, and characteristic of suffering; the pulse is feeble, and sometimes intermits; there is hiccup, and all the symptoms of ileus. The action of the muscles at the neck of the bladder is drawn into disorder, and the urine is retained. This, which may be denominated simple stricture, is, Mr. Bell thinks, the consequence of inflammation in the gut, excited by frequent ineffectual efforts to propel the fæces in a constipated state of the bowels.

"The sphincter in this condition does not relax, nor does the intestine itself act. The whole propelling power is in the abdominal muscles. The rectum, urged down by the pressure from above, forms a fold of the inner coat, just above the inner sphincter. By repetition, inflammation and adhesion of the outer sides of the fold take place; and by these means losing its softness and yielding nature, it becomes a permanent septum, standing nearly across the gut. It will be removed with difficulty, in proportion to the time that has elapsed from its formation." 314.

The means of cure recommended by our author are, proper laxatives regularly taken, the use of glysters to prevent the lodgment of fæces above the stricture, and the daily introduction of the bougie or tent. The division of the stricture with the knife should not be thought of till other means have failed; and when done, it requires the use of the bougie to perfect the cure.

"To cut the stricture, we take the probe-pointed bistoury, used for hernia, which has a cutting edge, of only about half an inch in length, near its extremity. Having introduced the tip of the finger into the stricture, so as to feel the firm edge distinctly, the knife is laid with its flat side upon the finger, and pushed along under its guidance, until the end enters the stricture. The cutting edge of the knife is then to be turned round, so as to present the edge to the stricture; and it is to be urged against the stricture by the finger in ano. The cut being made on the edge of the stricture in one direction, it is better to turn the knife flat upon the finger, and then to move both the finger and knife round to some other part of the edge of the stricture, which stands most distinct and prominent; and thus to notch the membrane in several places. This will be more effectual, and attended with less hazard, than one deep cut." 315.

The *scirrhous* contraction of the rectum is an incurable disease. It begins with a hard tumour, smooth towards the cavity of the gut, attended by shooting pain or constant uneasiness, and difficulty of passing the fæces. In a few weeks the tumour becomes irregular, occupying one side of the gut, but gradually spreading all around it. The finger cannot now be introduced; the evacuations are much obstructed, and attended with burning pain; the patient cannot sit, and the spasmodic retraction of the anus, over which he has no control, gives him insufferable pain. Lodgments of indigestible substances above the stricture are now apt to induce inflammation and abscess. Here, while relating a most interesting case of this deplorable malady, Mr. Bell takes occasion to remind his readers that indigestible matters, as hard bones, may pass through the whole line of canal, until they are retained by the sphincter ani, where they fix themselves, irritating and penetrating the coats of the intestines, and causing great alarm of a cancerous disease.

When a cancerous affection is ascertained, our hopes must rest on soothing the general irritability, and preserving the diseased parts as quiet as possible. A gentle laxative may be joined with cicuta, or the castor oil in conjunction with laudanum, or extract of hyoscyamus with extract. col. comp. and after every motion a tepid glyster should be thrown up. The rectum should be kept free for the passage of fæces, by means of the tent; and if there be excessive irritation from the fæces coming in contact with the ulcerated surface of the gut, the elastic tube must be used to draw them off. Leeches, fomentations, and opiate glysters, will be occasionally required.

4. *Prolapsus ani* is generally a consequence of irritation in the rectum, and not of relaxation. It is frequent in children, from the nestling of ascarides in the gut. The irritation of these worms produces a perpetual nisus in the intestine, until it at last acts upon itself, and the inner membrane is protruded. It swells, congestion takes place in the parts, and the intestine is pushed further and further out. A long-continued use of irritating purges will produce the same effect in children, and even in adults.

"To reduce the prolapsus, we must order fomentation to the protruded parts: after this, we undertake a general and gradual compression of the protruded gut. When the bulk is diminished, we then attempt to reduce the gut, and place it within the anus. In children, it is difficult to reduce the last turns, if the finger is pushed through the orifice; for when it is again withdrawn, the gut slips down. We may, therefore, twist a piece of stiff paper into the form of a cone, soften the point by wetting it, and oil the surface of the paper: placing this upon the point of the finger, with it we may push the last portion of the gut within the anus; the cone will slip out easily, without bringing down the gut with it." 326.

The cause, however, still remains to be removed—the irritation and tenesmus. If worms are the cause, bitter infusions or lime water should be thrown up. Other sources of irritation must be sought for, in some cases, and the cure will be assisted by injection of starch and laudanum; and by washing the parts with an infusion of galls and opium, when they are protruded. The child should not be permitted to strain, nor to take the usual position at stool. He should be kept in the erect posture, and the hips should be held together, so as to compress and support the gut.

*Chronic Prolapsus.* This is quite a different disease from the one just described. It is a protrusion of the inner fold of the rectum, the consequence of long-continued or habitual costiveness. When the torpor of the bowels has continued a long time, the protruded fold of the rectum becomes elongated, and assumes the form of a tumour, when inflamed and turgid; but of a shrivelled membrane, when not swollen and inflamed. In its ordinary condition, it presents two distinct membranous folds, one hanging from each side of the anus. Piles are often excited by, and added to this disease. "It is now that we, with propriety, cut off these pendulous flaps of skin."

"I have done this operation by taking hold of one of the flaps, with my finger and thumb, and passing the needle through it near its base, then cutting the ligature so as to make two: these being tied, one on each side of the flap, the projecting part is snipped off with the curved scissars. The ligature and remaining part of the flap are left to be withdrawn within the gut. A starch clyster, with laudanum, should be given in the evening, and the patient must keep to his bed or sofa for several days." 328.

5. *Hæmorrhoids.* Mr. Bell and Mr. Shaw make many judicious observations on hæmorrhoids, as we know from painful personal experience. Mr. Bell most wisely views piles as something more than a mere tumefaction of the hæmorrhoidal vessels, or inner membrane of the rectum. Irregularity of action in the small or great intestines will cause piles, but disordered secretion in the liver is most commonly the cause. As preventives, therefore, we have found nothing so effectual as the quicksilver pill and ipecacuanha, with the use of injections, so as to bring off the fæces without irritation. The introduction of the rectum bougie we have lately found very useful; and we have thought that the action of the bougie on the mucous membrane of the rectum has, in our own case at least, produced an increased action or secretion throughout the whole line of the alimentary canal, with an improved digestion. But this observation rests only on personal feelings in a solitary instance, and wants confirmation.

Mr. Bell cautions his reader, 1st. against including the fine skin at the margin of the anus in his ligature, when he proceeds to extirpate a pile. 2d. Never to leave the tumour strangulated by the ligature, and liable, by more distention, to draw in the surrounding skin. 3d. When he has drawn the ligature, to cut off all the convexity of the tumour with scissars. 4th. To avoid operating on a tumour which is broad and tense at its base. The following is Mr. Bell's mode of operating on hæmorrhoidal tumours.

"The patient resting on his knees, the assistant holds aside the nates. The surgeon taking hold of the tumour betwixt his finger and thumb, draws it down so as to expose the base of it. Now let him pass the hair-lip pin across the base of the tumour; take off the steel point from the silver pin. Over the pin, and consequently fully over the tumour, he is now to throw his ligature. He is to draw it as much as the patient can bear without excessive pain. With one motion of the long curved scissars he is to remove the tumour, which is thus included in the ligature." 336.

Mr. B's object, in the first part of the operation, is to restrain the bleeding, and to keep the membranes in contact, that they may adhere and be consolidated. On the succeeding morning, or in the evening of the day of operation, the pin may be withdrawn, if there be tension and pain of the part, for then its purpose is served. But, in general, the pin and the ligature may be permitted to remain until the parts go through the whole process of inflammation.

6. *Fistula in Ano.* This is a consequence of inflammation and suppuration by the side of the lower part of the rectum. But these are of different kinds, and require discrimination. One is a superficial phlegmonous boil in the skin, near the anus, from cutaneous irritation. In another person, of bad constitution, an abscess will take place in the loose cellular membrane at the side of the gut, ushered in by fever and restlessness, and exhibiting a dusky red inflammation, or perhaps a purplish colour, without much phlegmonous swelling. A bad suppuration follows, and the thin cellular membrane soon becomes sloughy. In a third person, particularly in advanced age, and broken constitution, a kind of carbuncle takes place, and degenerates into a fistula. In a fourth, the true chronic abscess will appear by the side of the anus, having a remote source. It appears, and becomes prominent without pain, or tension, or inflammation of the part, and is a mere consequence of the infiltration and gravitation of pus, from a disease high in the spine, perhaps of the nature of psoas abscess. Fifthly, in consumptive and scrofulous habits, abscesses will form by the side of the anus, upon very slight local irritation, and become in the end, if not from their commencement, constitutional. Here Mr. Bell draws the attention of his brethren to abscess formed by the side of the rectum, from irritation, or disease within the passage, a circumstance rarely noticed by surgical writers. Yet many analogies would lead us to think that there was nothing extraordinary in this. We have abscess external to the fauces, from inflammation of the inner membrane of the throat—abscess by the side of the lacrymal duct, and by the side of the urethra, in the perineum, from irritation and inflammation existing within these tubes. In fact, "we must consider that the cellular membrane is many degrees more disposed to the formation of abscess than any other texture; and as an inflamed gland, itself free from suppurative action, will yet produce pus in the surrounding cellular membrane, so the extremity of the rectum, without being properly the seat of suppuration, will yet cause it in the loose texture which is around it."

This view leads to a practical principle. Besides the usual remedies for bringing a suppuration to a successful issue, we must keep our attention on the state of the gut. If there be piles, warts, strictures, tenesmus, irritation, or discharge, they must be removed; for they have produced the abscess, and will cause its continuance. An abscess thus generated, is like a wound with a foreign matter in it. It becomes a sinus—its sides are consolidated—it discharges a thin fluid, and has no disposition to heal. At last the gut ulcerates, fæculent matter gets into the sinus, and true fistula is completed.

Mr. Bell lays down concise, yet clear and judicious, directions for operations necessary in cases of fistulæ in ano, but they do not differ materially from what are stated in our best systematic works of surgery.

The work closes with an Appendix, containing the description of the preparations which are in the fourteenth division of the Museum in Windmill Street, and exhibits a vast mass of important pathological specimens, well worthy the attention of students, and all visitors who can get access to this invaluable collection.

Our readers will perceive that our analysis only embraces the two wings of the work under Review. The middle, and great body of the publication, on stricture, its consequences, and concomitants, being passed over, at least for the present, as not within the grasp of even a very extended article. From the specimens of valuable matter which we have exhibited in this analysis, our readers will easily form an estimate of the work, which we most earnestly recommend to the attentive perusal not only of the surgeon and general practitioner, but of the physician. One great charm of Mr. Bell's writings consists in his very ingenious physiological and pathological elucidations of disease, equally interesting to the medical and chirurgical practitioner. Another is the force with which he impresses all important practical principles on the attention, and consequently on the memory of his readers or auditors. A third, is the air of simplicity and perspicuity which he manages to throw over the most intricate and difficult subjects, by which the student is encouraged to proceed in his investigations, instead of being checked and embarrassed by awkward and unsatisfactory attempts at the explanation of natural and morbid phenomena. These are great gifts, and indications of great talent. They are not entirely free from alloy. Mr. Bell does not pay much attention to composition and arrangement. Yet we think that these, however inferior in themselves, would add not a little to the value, as well as the lustre of Mr. Bell's publications. We may again state, that Mr. Shaw's notes and illustrations are exceedingly creditable to the professional zeal and acquirements of this deserving young surgeon.

